# Mitochondrial control region I and microsatellite analyses of endangered Philippine hornbill species (Aves; Bucerotidae) detect gene flow between island populations and genetic diversity loss

**DOI:** 10.1186/1471-2148-12-203

**Published:** 2012-10-12

**Authors:** Svenja Sammler, Valerio Ketmaier, Katja Havenstein, Ulrike Krause, Eberhard Curio, Ralph Tiedemann

**Affiliations:** 1 Institute for Biology and Biochemistry, Unit of Evolutionary Biology/Systematic Zoology, University of Potsdam, Karl-Liebknecht-Str. 24-25, Haus 26, 14476, Potsdam, Germany; 2 Department of Biology and Biotechnology “C. Darwin”, University of Rome “La Sapienza”, V.le dell’Universita’ 32, 00185, Rome, Italy; 3 Ruhr-University Bochum, Faculty of Biology and Biotechnology, Conservation Biology Unit, Universitätsstr. 150, 44801, Bochum, Germany

**Keywords:** Biogeography, Bucerotidae, Conservation genetics, Genetic diversity loss, Microsatellites, Mitochondrial control region I, Philippine archipelago, Phylogeography

## Abstract

**Background:**

The Visayan Tarictic Hornbill (*Penelopides panini*) and the Walden’s Hornbill (*Aceros waldeni*) are two threatened hornbill species endemic to the western islands of the Visayas that constitute - between Luzon and Mindanao - the central island group of the Philippine archipelago. In order to evaluate their genetic diversity and to support efforts towards their conservation, we analyzed genetic variation in ~ 600 base pairs (bp) of the mitochondrial control region I and at 12–19 nuclear microsatellite loci. The sampling covered extant populations, still occurring only on two islands (*P. panini*: Panay and Negros, *A. waldeni*: only Panay), and it was augmented with museum specimens of extinct populations from neighboring islands. For comparison, their less endangered (= more abundant) sister taxa, the Luzon Tarictic Hornbill (*P. manillae*) from the Luzon and Polillo Islands and the Writhed Hornbill (*A. leucocephalus*) from Mindanao Island, were also included in the study. We reconstructed the population history of the two *Penelopides* species and assessed the genetic population structure of the remaining wild populations in all four species.

**Results:**

Mitochondrial and nuclear data concordantly show a clear genetic separation according to the island of origin in both *Penelopides* species, but also unravel sporadic over-water movements between islands. We found evidence that deforestation in the last century influenced these migratory events. Both classes of markers and the comparison to museum specimens reveal a genetic diversity loss in both Visayan hornbill species, *P. panini* and *A. waldeni*, as compared to their more abundant relatives. This might have been caused by local extinction of genetically differentiated populations together with the dramatic decline in the abundance of the extant populations.

**Conclusions:**

We demonstrated a loss in genetic diversity of *P. panini* and *A. waldeni* as compared to their sister taxa *P. manillae* and *A. leucocephalus*. Because of the low potential for gene flow and population exchange across islands, saving of the remaining birds of almost extinct local populations - be it in the wild or in captivity - is particularly important to preserve the species’ genetic potential.

## Background

The Philippine archipelago consists of more than 7,000 islands, mostly of volcanic origin, with a well known geological and plate tectonic history [1]. According to various authors e.g. [2-6], during the Pleistocene glaciations, the Philippine islands were less fragmented than today; groups of islands formed composite Pleistocene Aggregate Island Complexes (PAICs) [5], that were isolated from one another by deep-water channels. Apart from Greater Palawan, which, biogeographically, does not belong to the oceanic region of the Philippines [4], there were three major PAICs: Greater Luzon, Greater Negros-Panay, and Greater Mindanao [3]. The geological history has had a great impact on the evolution of the Philippines’ fauna and flora [7] resulting in an extremely high rate of over 57% endemism in the major faunal groups [8], with many lineages limited to single islands or PAICs [4]. Among Philippine vertebrates, this is especially true for reptiles, amphibians, and mammals. In contrast, birds show, due to their volant nature, relatively low levels of endemism. Here, dispersal may be thus more an important driver of species diversity than vicariance [9].

Because of their species richness, the Philippines belong to the 25 biodiversity hotspots on earth, but are simultaneously the one with the lowest percentage (3%) of remaining primary vegetation [10]. According to satellite data from 1987, forest cover has declined to 23.8%, but varies considerably across the archipelago [11]. For example, Luzon retained 24% forest cover and Mindanao 29%; in the Western Visayas, Panay retained 8% and Negros 4%. Even these low numbers are probably overestimates, as only a proportion comprises closed-canopy forest [11,12]. In 2002, estimates for the whole archipelago of 21.7% and 24.4% (depending on source) forest cover reflect some slowing down in the rate of deforestation [13,14]. The remaining forest is of critical importance for the survival of many threatened species, including the hole-nesting hornbills (Bucerotidae), which, due to their size, depend on primary or old secondary growth for their reproduction.

The distribution of hornbills in the Philippines largely corresponds to the composition of the PAICs. These birds are a prominent faunal element of the Archipelago such that, based on the occurrence of the different hornbill species, Kemp [15] divided the Philippines into six zoogeographical regions, the Luzonian, the Mindoroan, the Panay-Negrosian, the Samarian the Mindanaoean, and the Suluan, corresponding to the three major and two smaller PAICs (according to Heaney [2], the Samarian and the Mindanaoean formed one PAIC).

**Figure 1:**
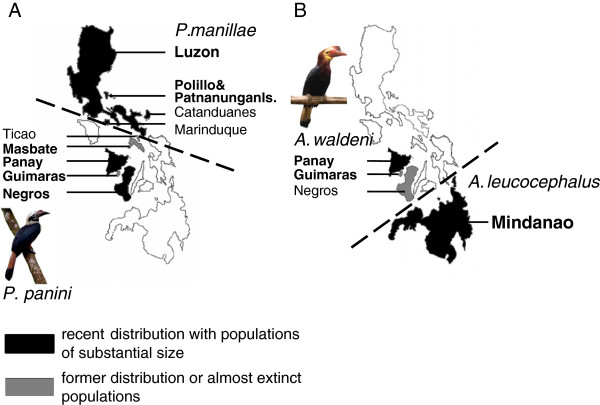
**Former and recent distribution of (A) *Penelopides manillae *and *P. panini *and of (B) *Aceros waldeni *and *A. leucocephalus. *** The dashed lines approximately delineate the distribution ranges of the respective sister species. The illustration of the Philippines is restricted to larger islands and islands with former or recent occurrence of the studied hornbill species. Labeled islands are mentioned in the text. Sampled populations are indicated by island names in bold face.

The focal species of this study, the Visayan Tarictic Hornbill (*Penelopides panini*) and the Walden’s Hornbill (*Aceros waldeni*), occur in the Panay-Negrosian region (Greater Negros-Panay, Figure 1), the most threatened faunal region of the Philippines [16]. For comparison, their less endangered (= more abundant) respective sister taxa, the Luzon Tarictic Hornbill (*P. manillae*) from the Luzonian (Greater Luzon) and the Writhed Hornbill (*A. leucocephalus*) from the Mindanaoean (Greater Mindanao) are included in this study [15].

*A. waldeni* and *P. panini* are endemic to the western islands of the Visayas, which constitute - between Luzon and Mindanao - the central island group of the Philippine archipelago. Due to deforestation and hunting, their populations decreased rapidly [12]. *P. panini* is known from Panay (including offshore islands), Guimaras, Negros, Masbate, and Ticao Island (here with a second recognized subspecies *P. p. ticaensis*[17,18]). Today, it is still fairly common on Panay, less so on Negros, and extinct, or nearly so, on all other islands (Figure 1A) [19-21], EC pers. obs. Recently, a remnant population of three birds was found in Milagros, Masbate, but this very small native forest patch is slowly being converted into agricultural land, rendering the survival probability of this remnant population very low [22]. *P. panini* is listed as endangered by BirdLife International [12].

*A. waldeni* used to occur on three islands, Panay, Guimaras, and Negros (Figure 1B) [18], but has survived with a breeding population of substantial size only on Panay, in the forest of the Central Panay Mountain Range [23]. It is extinct on Guimaras and may be extinct on Negros as well, as no recent sightings have been reported [20], EC pers. obs. It is listed as critically endangered by BirdLife International [12].

Captive breeding of both threatened Visayan hornbill species is very rare and incipiently. The world’s only captive population of *A. waldeni* (22 individuals) is held in a breeding facility on Panay. In 2005, for the first time, a total of three offspring were successfully bred by two pairs [24]. *P. panini* have bred there successfully since 2003 [24]. Former captive reproduction success within the genus *Penelopides* could have also include *P. panini*, but due to uncertainty about species level in this genus [15,18], the different species most probably had been hybridized in captivity [25].

The less endangered *P. manillae* is classified into two subspecies, separated morphologically by size, the width of the tail band and the glossiness of the upperparts [15,18,26]; *P. m. manillae* occurs on Luzon and smaller adjacent islands, such as Marinduque and Catanduanes, *P. m. subniger* on Polillo and Patnanungan Islands (Figure 1A) [18,26-28]. BirdLife International [12] lists the species in the category ‘least concern’.

The less endangered *A. leucocephalus* occurs on Mindanao and its satellite islands (Figure 1B) [18]. Its conservation status is poorly known, but it is considered locally fairly common in suitable habitat and is listed as near threatened by BirdLife International [12].

In order to support conservation efforts for Philippine hornbill species, an assessment of the genetic population structure of the remaining wild populations is a basic requirement. Not many population genetic studies have so far been carried out in the Philippines; this is especially true for birds. Patterns of colonization and diversification in Passeriformes have been studied in a phylogeographic context by Jones and Kennedy [7,29] and Silva-Iturriza et al. [30], and at higher taxonomic levels by Oliveros and Moyle [9] and Sánchez-González and Moyle [31]. Here, we reconstruct the phylogeographic history of the two *Penelopides* species in order to relate it to the geological history of the Philippines. We also give estimates on the genetic diversity both in the endangered species (*A. waldeni* and *P. panini*) and their less threatened sister taxa (*A. leucocephalus* and *P. manillae*) in order to test, whether the decline in population sizes in the endangered species is also reflected in lower genetic diversity.

## Methods

### Sampling and DNA extraction

One drop of blood was taken from captive hornbills and stored in Queen’s Lysis Buffer [32]. The number of sampled individuals per population, their origin and abbreviation used in the following text and figures are given in Table 1. In addition, 15 feather samples of *P. panini* and 7 feather samples of *A. waldeni* were collected from molted feathers in nest holes of wild birds on Panay. Furthermore, 24 museum samples from The Natural History Museum, Tring (U.K.; BMNH) and the Museum für Naturkunde, Berlin (Germany; ZMB) - all dating back to the 19^th^ century - were included in the study (Table 2).

**Table 1:** Blood samples of captive hornbills included in the study

**Number of samples**	**Species**	**Origin**	**Abbreviation in text**
62	*P. panini*	Panay (confiscated from poaching → of wild origin)	PpPa
14	*P. panini*	Negros (Bacolod City Biodiversity Conservation Center of the Negros Forest and Ecological Foundation, Inc./Ecological Park, Dumaguete)	PpNe
17	*P. manillae manillae*	Luzon* (Birds International, Inc., Quezon City/Malagos Garden Resort, Davao City/Avilon Montalban Zoological Park, Rizal)	Pmm
9	*P. manillae subniger*	Polillo* (Birds International, Inc., Quezon City)	Pms
22	*A. waldeni*	Panay (confiscated from poaching → of wild origin)	Aw
4	*A. leucocephalus*	Mindanao (Malagos Garden Resort, Davao City/Avilon Montalban Zoological Park, Rizal)	Al

* origin concluded from morphological determination of captive individuals.

**Table 2:** Museum specimens included in the study. BMNH, The Natural History Museum, Tring (U.K.); ZMB, Museum für Naturkunde, Berlin (Germany)

**Voucher**	**Genus**	**Species**	**Subspecies**	**Island, locality**	**Collecting date**	**GenBank acc. no.**
BMNH 1896.4.15.67	*Penelopides*	*panini*	*panini*	Guimaras	6 Jan 1888	JX273923
BMNH 1896.4.15.68	*Penelopides*	*panini*	*panini*	Guimaras	28 Dec 1887	JX273924
BMNH 1896.4.15.69	*Penelopides*	*panini*	*panini*	Guimaras	23 Jan 1888	JX273925
BMNH 1888.10.30.99	*Penelopides*	*panini*	*panini*	Guimaras	Mar 1872	JX273926
BMNH 1888.10.30.100	*Penelopides*	*panini*	*panini*	Guimaras	Mar 1872	JX273927-JX273928
BMNH 1888.10.30.101	*Penelopides*	*panini*	*panini*	Guimaras	Mar 1872	JX273929
BMNH 1888.10.30.102	*Penelopides*	*panini*	*panini*	Guimaras	Mar 1872	JX273930-JX273931
ZMB 21775	*Penelopides*	*panini*	*panini*	Guimaras	Mar 1872	JX273932
BMNH 1896.4.15.70	*Penelopides*	*panini*	*panini*	Masbate	1 May 1888	JX273933
BMNH 1896.4.15.71	*Penelopides*	*panini*	*panini*	Masbate	1 May 1888	JX273934
BMNH 1896.4.15.72	*Penelopides*	*panini*	*panini*	Masbate	1 May 1888	JX273935
ZMB 9641	*Penelopides*	*manillae*	*manillae*	Luzon, Manila	1830-1832	JX273954
ZMB 21772	*Penelopides*	*manillae*	*manillae*	Luzon, around Manila	Jan 1872	JX273955
ZMB 21773	*Penelopides*	affinis*	affinis*	Luzon, around Manila*	Feb 1872	JX273975
ZMB 21774	*Penelopides*	*manillae*	*manillae*	Luzon, around Manila	Jan 1872	JX273956-JX273957
ZMB 32616	*Penelopides*	*manillae*	*manillae*	Culion, Palawan? (see discussion)	?	JX273958-JX273959
ZMB 2000.22207	*Penelopides*	*manillae*	*manillae*	Luzon, Manila	4-9 Jun 1846	JX273960
ZMB 2000.22208	*Penelopides*	*manillae*	*manillae*	Culion, Palawan? (see discussion)	?	JX273961-JX273962
BMNH 1896.4.15.98	*Aceros*	*waldeni*		Guimaras	28 Jan 1888	JX273808
BMNH 1896.4.15.99	*Aceros*	*waldeni*		Guimaras	28 Dec 1887	JX273809
BMNH 1896.4.15.100	*Aceros*	*waldeni*		Guimaras	27 Dec 1887	JX273810
ZMB 2000.22212	*Aceros*	*waldeni*		Guimaras	6 Jan 1888	JX273811
ZMB 2000.22215	*Aceros*	*waldeni*		Guimaras	Jan 1888	JX273812
BMNH 1897.5.13.471	*Aceros*	*waldeni*		Negros	27 Mar 1896	JX273813

* for a note on taxonomic status and origin stated on the museum label of ZMB 21773 see Additional file 1.

DNA extraction of blood samples was performed using the DNeasy Tissue Kit (Qiagen) according to the manufacturer’s instructions for blood samples. Feather samples were digested with 15 μl proteinase K (20mg/ml) in the presence of 40 μl DTT and 5 μl Carrier RNA (1 μg/μl) in 450 μl buffer solution (100 mM Tris, 10mM EDTA, 100mM NaCl, 0.1% SDS). After incubation at 55°C over night, we added again 15 μl proteinase K (20mg/ml) and incubated the mix for one more hour. Afterwards, we purified the samples according to the manufacturer’s instructions of the DNeasy Tissue Kit (Qiagen), but increased EtOH to 400 μl and the washing solutions to 600 μl. DNA was eluted twice in 50 μl of the buffer solution provided with the kit.

DNA extraction from toe-pad samples of museum specimens were performed using the DNeasy Tissue Kit (Qiagen), according to the manufacturer’s instructions, adding 5 μl Carrier-RNA (1 μg/μl). In all steps, solution volumes were doubled. DNA was eluted twice, initially in 50 μl and then in 30 μl of the kit buffer solution. DNA extraction from museum samples was carried out in a dedicated ancient DNA laboratory, physically separated from the laboratory where contemporary DNA was handled.

### mtDNA amplifying and sequencing

The mitochondrial genome of Philippine hornbills contains two control regions of which we amplified the 5’end of the first control region (CRI) as described for fragment 3 in Sammler et al. [33]. Products were sequenced with primer AcePen_Glu-for to obtain the targeted first ~ 650 base pairs.

Since the amount of DNA isolated from molted feathers and museum specimens is low and the DNA often degraded, the corresponding sequences of these samples were amplified in two short, partially overlapping fragments. The first fragment was amplified with the forward primer AcePen_Glu-for and a CRI specific reverse primer (Penpan_CRI-spec-180rev or PenpanGui_CRI-spec-182rev for *P. panini*, Penmanman_CRI-spec-185rev for *P. m. manillae*, Penaff_CRI-spec-186rev for museum specimen ZMB 21773, and Acewal_CRI-spec-180rev for *A. waldeni*). The second fragment was amplified with a CRI specific forward primer (Penpan_CRI-spec-128for for *P. panini*, Penmanman_CRI-spec-115for for *P. m. manillae*, Penaff_CRI-spec-124for for museum specimen ZMB 21773, and Acewal_CRI-spec-130for for *A. waldeni*) and the reverse primer AcePen_644rev or, when amplificates did not yield sufficient results, also with AcePen_426rev (Table 3).

**Table 3:** PCR primers used to amplify CRI gene fragments. Y = C/T; R= A/G

**PCR primers**	**Primer sequence (5′-3′)**	**Reference**
AcePen_Glu-for^a^	GCT TTT CTC CAA GGT CTA CAG CTC	[33]
AcePen_Cyt1018-rev^a^	GGG TGT TCT ACT GGT TGG CTG CC	[33]
Penpan_CRI-spec-180rev	CRY YRT YYA CAT TAA GTG A	This study
PenpanGui_CRI-spec-182rev	GTC ATT ATC TAC ATT AAG TAA G	This study
Penmanman_CRI-spec-185rev	TTA ATA TGT CGT TGT TTA CAT G	This study
Penaff_CRI-spec-186rev	GTA TGT CAT TGC TTG CAT TGA GTA G	This study
Acewal_CRI-spec-180rev	CAT TRT CTG CAT TTA AGC GT	This study
Penpan_CRI-spec-128for	ACG ACT AGT TAT TAA TGC T	This study
Penmanman_CRI-spec-115for	CAT AAG GTA ATG CTC TAT ACA ATT	This study
Penaff_CRI-spec-124for	CTA TAT GAT TAA CTA TTA ATG CTC	This study
Acewal_CRI-spec-130for	GAT TGA CTG TCA ATG TTT GT	This study
AcePen_644rev^a^	AAG GGA ACC AAC AGT GCC AAA C	This study
AcePen_426rev^a^	GTT GCT GAT TTC TCG TGA GG	This study

^a^ primers with two annealing sites due to the duplication of a large fragment of the hornbills’ mt genome [33].

The 37.5 μl PCR reaction volumes (buffer solution: 10mM Tris-HCl, pH 9.0, 50mM KCl, 1.5mM MgCl_2_, 0.1% Triton X100, 0.2 mg/ml Bovine Serum Albumin (BSA)) were set up as follows: 6 μl dNTP Mixture (2.0 mM each), 1.2 μl of each primer (10 μM), 3 μl DNA template, 0.25 μl *Taq* polymerase (5U/μl, MP Biomedicals). The reaction was performed under the following conditions: initial denaturation at 94°C for 5 min; 50 cycles: 94°C for 90 s, 50°C for 1 min, 72°C for 1 min; and a final extension at 72°C for 10 min. Products were sequenced as described in Sammler et al. [33] with AcePen_Glu-for, AcePen_644rev or AcePen_426rev, respectively.

To verify the authenticity of amplificates from historical samples, all the mtDNA PCRs of museum samples were repeated by another person in a separate laboratory in a plant genetics department, where animal DNA had never been handled. Specifically, all repeated PCR/sequencing analyses of the first fragment yielded identical results in 1^st^ and 2^nd^ PCR. For the second fragment, 1^st^ and 2^nd^ PCR also yielded identical results, except for two occasions with a single bp mismatch. Scarcity of DNA precluded a third PCR, but these two mismatches translate into an per base pair error rate of about 0.014%, a measure in the range of known *Taq* error rates. There was no indication of an elevated error rate in the historical samples.

Sequences were aligned in BioEdit version 7.0.5.3 [34]. Mitochondrial haplotypes were defined based on a 591–593 bp data partition. Distance matrices and statistical parsimony haplotype networks were constructed using TCS 1.21 with default parameter settings, but a fixed connection limit at 100 steps [35].

### Microsatellite genotyping

All blood samples were genotyped at 12–19 previously published polymorphic microsatellite loci (Table 4). PCR and fragment size analysis of listed loci were carried out as described in Sammler et al. [36]. Annealing temperatures of Bbi and Bubi primers were set to 55°C.

**Table 4:** Microsatellite loci used in the study

**Loci**	**Genotyped species**	**Reference**
Bbi2	*P. panini*, *P. manillae*, *A. waldeni*, *A. leucocephalus*	[37]
Bbi7	*P. panini*, *P. manillae*, *A. waldeni*, *A. leucocephalus*	[37]
Bbi13	*P. panini*, *P. manillae*	[37]
Bbi16	*P. panini*, *P. manillae*, *A. waldeni*, *A. leucocephalus*	[37]
Bubi294	*P. panini*, *P. manillae*, *A. waldeni*, *A. leucocephalus*	[37]
Bubi326	*P. panini*, *P. manillae*, *A. waldeni*	[37]
Pp_GA_3	*P. manillae*	[36]
Pp_GA_4	*P. panini*, *P. manillae*	[36]
Pp_GT_1	*P. panini*, *P. manillae*, *A. waldeni*, *A. leucocephalus*	[36]
Pp_GT_3	*A. leucocephalus*	[36]
Pp_GT_4	*P. panini*, *P. manillae*, *A. waldeni*, *A. leucocephalus*	[36]
Pp_GT_5	*P. panini*, *P. manillae*	[36]
Pp_GT_6	*P. panini*, *P. manillae*, *A. waldeni*, *A. leucocephalus*	[36]
Pp_GT_7	*P. panini*, *P. manillae*, *A. waldeni*, *A. leucocephalus*	[36]
Pp_GT_10	*P. panini*, *P. manillae*, *A. waldeni*	[36]
Pp_GT_17	*P. panini*, *P. manillae*	[36]
Pp_GT_18	*P. panini*, *P. manillae*, *A. waldeni*, *A. leucocephalus*	[36]
Pp_GT_19	*P. panini*, *P. manillae*, *A. waldeni*, *A. leucocephalus*	[36]
Pp_GT_21	*P. panini*, *P. manillae*, *A. waldeni*, *A. leucocephalus*	[36]
Pp_GT_22	*P. panini*, *P. manillae*	[36]

### mtDNA data analysis

Standard measures of genetic diversity (haplotype diversity, HD [38]; nucleotide diversity, ND [39]) as well as of genetic divergence among island populations (fixation index, F_ST_[40]), were calculated as implemented in the software package ARLEQUIN version 3.5.1.2 [41]. When more than two populations were compared (i.e., *P. panini*), we performed an analysis of molecular variance (AMOVA) with the same software. Significance of F_ST_/AMOVA results was evaluated by permutation analysis (1,000 permutations). Diagnostic mutations of each island population were identified visually in the alignment. ARLEQUIN was also used to assess the fit of the demographic expansion model [42] to hornbill genetic variations (each population singularly). Mismatch distributions were used to fit the model of sudden demographic expansion. Goodness of fit was assessed by the sum of square deviations (SSD) between the observed and the expected mismatch and its significance (*P*-SSD) was determined by a parametric bootstrap with 10,000 replicates. Mismatch distributions were not calculated for *A. leucocephalus* because of the small sample size of the single population of this species included in the study.

### Microsatellite data analysis

The program FSTAT 2.9.3.2 [43] was used to calculate allelic richness (AR). F_ST_ values were calculated with ARLEQUIN. Significance of these values was evaluated by permutation analysis (1,000 permutations). Private alleles (PA) were assessed with the Excel Add-In software GENALEX 6 [44]. ARLEQUIN was used to calculate observed and expected heterozygosity (H_O_ and H_E_, respectively) and to test for deviation from Hardy-Weinberg equilibrium (HWE) for each locus in each population using Fisher’s exact test and the Markov chain method (1,000 demorization steps, 100 batches, with 10,000 iterations per batch set). Levels of significance of the HWE and linkage disequilibrium tests were Bonferroni corrected for multiple comparisons. A Bayesian clustering was performed using the software STRUCTURE version 2.3.2 [45]. Genetic subdivision was evaluated estimating the likelihood and sample composition of independent runs of subgroups (K = 1–5), assuming an admixture model, with a burn-in length of 100,000 and a data collection period of 900,000 iterations. To check for convergence of the Markov Chain parameters, ten replicate runs for each value of K were performed. The value of K best suited for the data was determined according to Evanno et al. [46] and Earl and von Holdt [47].

Demographic signatures of bottlenecks were investigated using the BOTTLENECK 1.2.02 software [48] under three different possible evolutionary models, i.e., Infinite Allele Model (IAM), Stepwise Mutation Model (SMM), and Two-Phase Model (TPM). Estimations of significant deviations from the null hypothesis of mutation-drift equilibrium were based on 10,000 replications.

## Results

### mtDNA diversity and divergence

#### Penelopides panini

**Figure 2:**
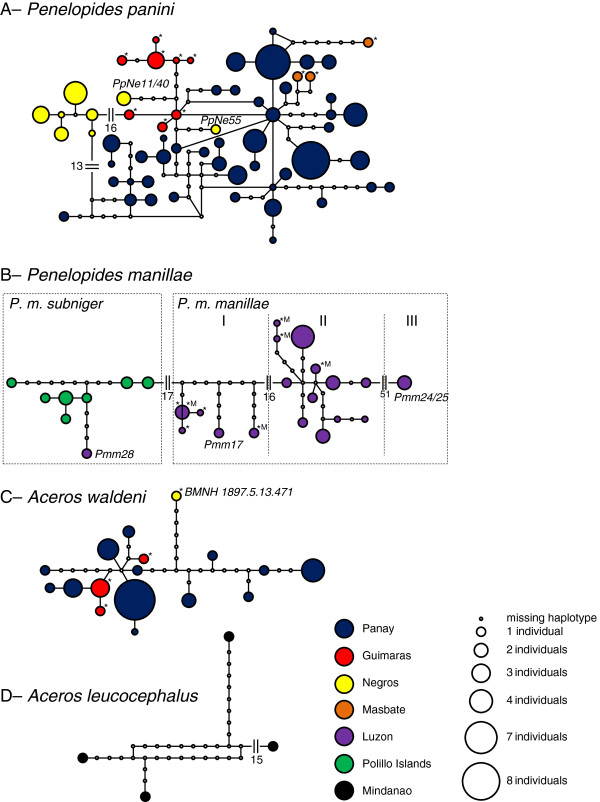
**Haplotype networks of (A) *Penelopides panini, *(B) *P. manillae, *(C) *Aceros waldeni, *and (D) *A. leucocephalus. *** For heteroplasmic individuals, each haplotype frequency was weighted by 0.5. Italic labels indicate individuals that are discussed in detail in the text. Haplotypes based on museum specimens are marked by an asterisk, those of *P. manillae* with known origin from the Manila area additionally by an M.

Based on the first 593 bp of the mitochondrial control region I sequences obtained for 102 specimens, we found 76 polymorphic sites (one indel, 75 transitions), defining 62 unique haplotypes (Figure 2A) [GenBank: HQ834451, HQ834457-HQ834461, JX273818-JX273935]. Due to single heteroplasmic sites cf. [33,49], 22 specimens exhibited two haplotypes. The frequency of such haplotypes was weighted by 0.5 in the frequency representation of Figure 2. The two most abundant haplotypes were found in 8 individuals each; all others are only represented by four or less individuals (Figure 2A). The AMOVA revealed a highly significant overall divergence among populations (F_ST_=0.572, p<0.001), such that 57% of the mtDNA variation was apportioned to divergence among populations. All single populations had significantly diverged from each others (pairwise F_ST_ between 0.284 and 0.664; p<0.01; see below for details). Three individuals originating from Negros (PpNe11, 40, 55) carry two unique haplotypes that differ by 18–24 substitutions from all other individuals coming from the same area. Both haplotypes are genetically more similar to haplotypes originating from Panay/Guimaras (4 and 5 substitutions away from the respective closest haplotype, Figure 2A). Apart from these three individuals, the two recent populations of Panay and Negros are clearly separated by at least 17 mutational steps in the network and seven diagnostic mutations in the alignment. The fixation coefficient (F_ST_) between these populations is 0.665 (p<0.001), but rises to 0.765 (p<0.001) when calculated excluding the three individuals exhibiting deviant haplotypes (Pp11/Pp40 and Pp55). Haplotype diversities range from 0.900 (Negros) to 1.000 (Masbate). The Negros population has a nucleotide diversity higher than that found on Panay (ND = 0.015 vs. 0.010, Table 5), but when the two exceptionally divergent haplotypes (PpNe11/40, PpNe55) are removed from computations, ND drops to 0.004. The highest number of base substitutions between two individuals of the species is 28, corresponding to a nucleotide divergence of 4.7%.

**Table 5:** mtDNA and microsatellite diversity measures

	***P. panini***	***P. panini***	***P. panini***	***P. panini***	***P. manillae manillae***	***P. manillae subniger***	***A. waldeni***	***A. waldeni***	***A. waldeni***	***A. leucocephalus***
	**(Panay)**	**(Negros)**	**(Guimaras)**	**(Masbate)**	**(Luzon)**	**(Polillo)**	**(Panay)**	**(Guimaras)**	**(Negros)**	**(Mindanao)**
n/H	77/43	14/8	8/6	3/3	23/19	9/7	29/13	5/3	1/1	4/4
HD	0.975±0.005	0.900±0.046	0.933±0.077	1.000±0.272	0.966±0.020	0.910±0.035	0.897±0.035	0.700±0.218		1.000±0.177
ND	0.010±0.006	0.015±0.008	0.008±0.005	0.014±0.011	0.037±0.019	0.007±0.004	0.011±0.006	0.005±0.004		0.038±0.026
DM	0	7^a^	0	0	7^b^		0	0	4	
loci	18	18			19	19	13			12
n	62	14			17	9	22			4
H_O_	0.586 ± 0.280	0.642 ± 0.236			0.547 ± 0.311	0.576 ± 0.337	0.496 ± 0.278			0.625 ± 0.271
H_E_	0.640 ± 0.239	0.665 ± 0.182			0.605 ± 0.280	0.578 ± 0.240	0.543 ± 0.236			0.645 ± 0.240
PA	2.111	0.611			3.789	0.737				
AR	5.07	4.78			5.20	3.53	2.88			3.91

*n* number of samples analyzed, *H* number of haplotypes, *HD* haplotype diversity (± standard deviation, SD); *ND* nucleotide diversity (± SD), *DM* diagnostic mutations, *H*_*O*_ mean observed heterozygosity (± SD); *H*_*E*_ mean expected heterozygosity (± SD), *PA* private alleles, *AR* allelic richness over all loci.

^a^ when PpNe55, 11, 40 excluded.

^b^ when Pmm28 excluded.

The museum specimens from Guimaras and Masbate are all represented by unique haplotypes that cluster closer to haplotypes from Panay than to those from Negros (Figure 2A). There are no diagnostic mutations in the alignment which would clearly separate them from the Panay population. However, individuals of those two populations do not cluster randomly between the Panay haplotypes, but form separate subclusters (Figure 2A), 1–7 and 4–7 mutational steps away from the next Panay haplotype. Fixation indices to Panay are lower (Panay/Guimaras 0.295, p<0.001; Panay/Masbate 0.284, p<0.001) than to Negros (Negros/Guimaras 0.640, p<0.001; Negros/Masbate 0.576, p<0.001). Mismatch distributions do not deviate from the purely demographic expansion model (0.288 ≤ *P*-SDD ≤ 0.773) with the only exception of the population from Negros (*P*-SDD = 0.048).

#### Penelopides manillae

Based on 591 bp sequence in 32 specimens, we found 98 polymorphic sites (1 transversion, 96 transitions, 1 site with both a transversion and a transition), defining 26 unique haplotypes (Figure 2B) [GenBank: JX273936-JX273974]. Due to heteroplasmic sites, seven specimens are assigned to two haplotypes.

No haplotype is dominant in frequency (Figure 2B). Apart from individual Pmm28, both subspecies, *P. m. manillae* und *P. m. subniger*, are separated by at least 18 base substitutions in the network and can be distinguished from one another by 7 diagnostic mutations in the alignment. The F_ST_ between the two subspecies is 0.502 (0.529 when excluding Pmm28; both values are significant, p<0.001). *P. m. subniger* has a lower haplotype and nucleotide diversity than *P. m. manillae* (HD=0.910, ND = 0.007 vs. HD=0.966, ND=0.037, Table 5). The highest number of base substitutions between two individuals of the species is 70 (11.8% sequence divergence).

In the network, *P. m. manillae* forms three haplogroups (I, II, III, Figure 2B) separated by large genetic gaps. The first (I) is formed by four museum specimens and Pmm17. Two of the museum specimens (ZMB 9641, ZMB 21772) come from the Manila area, the other two (ZMB 2000.22208, ZMB 32616) are labeled “Culion”, an island north of Palawan. Seventeen substitutions separate haplogroup I from the second one (II). This is formed by most of the individuals sampled, including two museum specimens (ZMB 21774, ZMB 2000.22207), which also come from the Manila area. The third haplogroup (III), formed by Pmm24/25, is separated by at least 52 substitutions from haplogroup II. This genetic divergence is by far larger than that between the two morphologically differentiated subspecies *P. m. manillae* and *P. m. subniger* (Figure 2B).

Similar to what was observed in *P. panini*, we found also in *P. manillae* one individual (Pmm28) carrying a haplotype that does not fit with its morphological identification. Pmm28 is assigned morphologically to *P. m. manillae*, but its haplotype clusters closer to those of *P. m. subniger* (4–11 base substitutions), while there are at least 21 substitutions between Pmm28 and the closest *P. m. manillae* haplotype. Mismatch distributions never deviate from the purely demographic expansion model (0.156 ≤ *P*-SDD ≤ 0.371).

#### Aceros waldeni

Based on 593 bp sequence of 35 specimens, we found 33 polymorphic sites (2 indels, 1 transversion, 30 transitions), defining 17 unique haplotypes (Figure 2C) [GenBank: HQ834450, HQ834452-HQ834456, JX273781-JX273813]. Due to heteroplasmic sites, four specimens are assigned to two haplotypes.

With nine individuals, one haplotype is the most abundant; all others are only represented by four or less individuals (Figure 2C). mtDNA diversity is lower in Guimaras (HD=0.700, ND=0.005) than in Panay (HD=0.897, ND=0.011). The highest number of base substitutions between two individuals is 16 (2.7% sequence divergence). The five museum specimens originating from Guimaras carry three unique haplotypes that cluster among the Panay haplotypes. There are no diagnostic mutations in the alignment which would separate the individuals from Panay and Guimaras; the fixation index is 0.084 (p=0.063). The haplotype of the single museum specimen originating from Negros (BMNH 1897.5.13.471) is separated from the next Panay haplotype by 9 mutational steps in the network (Figure 2C) and 4 diagnostic mutations in the alignment (Table 5). Mismatch distributions never deviate from the purely demographic expansion model (0.393 ≤ *P*-SDD ≤ 0.373).

#### Aceros leucocephalus

Based on 592 bp sequence in 4 specimens, we found 41 polymorphic sites (all transitions). All individuals bear unique haplotypes (Figure 2D) [GenBank: JX273814-JX273817]. HD is 1.000 and ND is 0.038, both values however associated with high uncertainty (as reflected in SD values), due to small sample size. The highest number of base substitutions between two individuals is 30 (5.1% sequence divergence).

### Microsatellite diversity and divergence

The characteristics of the 14 Pp loci and their usability for population genetics analyses are presented by Sammler et al. [36]. Six of the eight analyzed Bbi and Bubi loci [37] were polymorphic, at least in the genus *Penelopides*. Their number of alleles per locus varied from 1 to 8 in *A. waldeni*, 1 to 6 in *A. leucocephalus*, 2 to 13 in *P. panini*, and 2 to 19 in *P. manillae*. We confirmed that no linkage disequilibrium could be found between any pair of loci.

#### Penelopides panini

**Figure 3:**
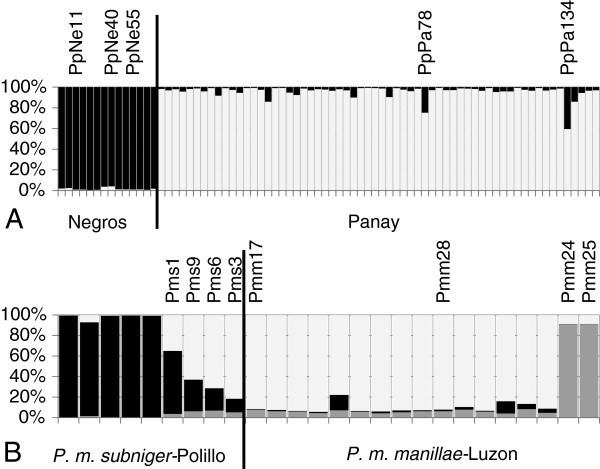
**STRUCTURE analysis of (A) *Penelopides panini *over 18 loci and of (B) *P. manillae *over 19 microsatellite loci. ** Each individual is represented by one vertical column divided into (**A**) K = 2 and (**B**) K = 3 colored clusters. Each color line is proportional to the individual’s estimated coefficient of membership to that particular cluster. Labeled individuals are discussed in detail in the text.

STRUCTURE results of independent runs with K ranging between 1 and 5 supported two as the most likely number of clusters. The clear division into two groups (Figure 3A) corresponds to the two populations from Panay and Negros. Intriguingly, the individuals carrying mtDNA haplotypes deviant from their region of origin (PpNe11/40, PpNe55; Figure 2A) are inconspicuous with regard to their microsatellite genotype. Interestingly, in two individuals from Panay (PpPa134 and PpPa78), about 25% of their genotypes is assigned to the Negros cluster.

The number of private alleles and the allelic richness over all loci is higher in the Panay than in the Negros population (PA = 2.11 vs. 0.61; AR = 5.07 vs. 4.78, Table 5) while this pattern is reversed for the observed and expected heterozygosities (Table 5). In the Negros population no loci deviate from the HWE while two loci are significantly out of HWE in the Panay population due to a lack of heterozygotes (data not shown). The fixation index between these two populations is 0.085 (p<0.001). For the Panay population, BOTTLENECK found a normal L-shaped allele frequency distribution as expected under mutation-drift equilibrium, suggesting that the effective population size has remained constant in the past [48]. For the Negros population, however, a shifted mode is indicative of a recent bottleneck.

#### Penelopides manillae

STRUCTURE was run with K = 1–5 and supports three as the most likely number of genetic clusters in this species. Individuals morphologically determined as *P. m. subniger* build a first cluster, and individuals of *P. m. manillae* build a second cluster, except Pmm24 and Pmm25, which group on their own (Figure 3B). Pmm24 and Pmm25 also had extremely divergent mitochondrial haplotypes (Figures 2B and 3). Four individuals (Pms1, Pms9, Pms6, Pms3) can be assigned partially both to the *subniger*- and the *manillae*-cluster. The individuals Pmm17 (haplogroup I of *P. m. manillae*; Figure 2B) and Pmm28, clustering closer to *P. m. subniger* in the mtDNA network (Figure 2B), do not stand out at the microsatellite genotypes, but are genetically similar to the other individuals assigned to *P. m. manillae* on morphological grounds.

The number of private alleles and the allelic richness over all loci is higher in *P. m. manillae* than in *P. m. subniger* (PA = 3.789 vs. 0.737; AR = 5.20 vs. 3.53, Table 5). H_O_ is nearly the same in the two subspecies while H_E_ is slightly higher in *P. m. manillae* than in *P. m. subniger* (Table 5); one locus deviates from HWE in either subspecies due to heterozygote deficiency (data not shown). The fixation index between these two populations is 0.050 (p<0.001). For *P. m. manillae*, computations with BOTTLENECK found a normal L-shaped allele frequency distribution as expected under mutation-drift equilibrium (constancy in the past population effective size) [48], whereas for *P. m. subniger* a shifted mode indicates a recent bottleneck.

#### Aceros waldeni

Analyses with STRUCTURE did not find any subpopulation structure in the sampling on Panay (K = 1 got best support; data not shown). H_E_ is slightly higher than H_O_ in the species (Table 5) and one locus shows deviations from HWE due to a lack of heterozygotes (data not shown). Computations with BOTTLENECK found a normal L-shaped allele frequency distribution as expected under mutation-drift equilibrium [48].

## Discussion

### *Penelopides panini*

Whereas the microsatellite data for *P. panini* show a clear division between the two populations from Panay and Negros (the sole exceptions being PpPa134 and PpPa78, Figure 3A), mtDNA revealed that three individuals from Negros (PpNe11, 40, 55) carry two haplotypes that are genetically more similar to haplotypes found on Panay, Guimaras and Masbate than they are to the remaining haplotypes found on Negros (Figure 2A). If we do not consider these two deviant haplotypes and the microsatellite genotypes of PpPa134 and PpPa78, the overall clear-cut subdivision in the genetic architecture of the species would be largely consistent with the geological history of the area. We hypothesize that substantial gene flow between Panay/Guimaras and Negros ceased at the end of the last Pleistocene ice age when the Panay-Negrosian PAIC broke down into separated islands due to eustatic changes in the sea level. While such a Pleistocene sea level-related model of speciation is rejected in favor of an earlier, Pliocene diversification for, e.g., rodents of the genus *Apomys*[50] or for the fruit bat *Haplonycteris fischeri*[51], the latter author suggests that sea-level changes may have played an important role for more recent divergence at the within PAICs level, such as among Negros and Panay. The shortest distance between the two islands today is about 15 km but, due to the presence of Guimaras and several other intervening islets, the longest open water distance is just 5 km. Tarictic hornbills are not strong flyers, and group-living, territorial species often make poor candidates for long-distance dispersal, even to offshore islands [52]. However, the three birds PpNe11, 40, 55 presumably descended from at least two female ancestors, which had recently, i.e., some generations ago, migrated from Panay or Guimaras to Negros. Since their haplotypes cluster relatively close to haplotypes from both islands (Figure 2A), the exact origin cannot be ascertained. Due to the general maternal inheritance of the mtDNA in vertebrates including birds (although, in rare occasions, paternal leakage has been found [53]), the haplotypes of these putative immigrants had persisted in the Negros population (Figure 2A), while most of their Panay/Guimaras ancestry has vanished at the microsatellite level due to the bi-parental inheritance of these markers (Figure 3A).

In the last 100 years, both Panay and Negros suffered from severe deforestation; Guimaras became completely denuded [11,14]. This complete deforestation might have forced birds from Guimaras to abandon the island and to seek still extant forested areas on Negros and Panay. These islands would then still host a (partial) mitochondrial heritage of Guimaras, although the maternal lineages carried by all 19^th^ century Guimaras museum specimens could not be found in our sampling. These Guimaras lineages have probably gone extinct. However, in this regard, a Guimaras origin of PpNe11/40, 55 seems the most plausible.

Today, the shortest distance between the remaining forest patches of Panay and Negros is about 100 km, a barrier that has become increasingly difficult for forest species to cross. As a consequence, we assume that gene flow between the two areas is currently strongly reduced or completely interrupted. The clear subdivision in the microsatellite data (Figure 3A) supports this hypothesis, the sole exceptions being PpPa134 and PpPa78.

Interestingly, for another forest bird species, the Philippine bulbul *Hypsipetes philippinus*, despite a small sample size of only three individuals from Negros, mtDNA suggested multiple independent colonization events for this island and a lack of a direct link between populations from Panay and Negros [30]. These authors, however, did not analyze any nuclear marker. A similar situation, again based only on mtDNA, was found for four out of the five passerine species studied by Jones and Kennedy [7,29]. However, in a fifth species, the Island Verditer-Flycatcher *Eumyias panayensis*, the populations of Negros and Panay are clearly separated and form sister clades [7]. Generally, dispersal across islands appears much more infrequent in our hornbill species than in the more vagile passerines.

Whereas the population of *P. panini* on Panay shows no indication of a recent bottleneck, the one on Negros does. This comes along with lower values of genetic diversity in the Negros population (Table 5). Negros retained half of the forest cover of Panay (4% vs. 8%) [11]; in addition, the forest cover on Panay (located mainly in the Central Panay Mountain Range) is almost continuous, while it is quite fragmented on Negros. We are aware that our sample size for this latter population is rather small for a precise estimate of its genetic diversity. We do not have access to 30 individuals, which is recommended for bottleneck analyses by Luikart et al. [54]. Small sample sizes are likely to miss alleles at low frequency [55] and might cause allele frequency distributions to resemble those of a bottlenecked population. Nonetheless, both our genetic analysis and the ecological factors are consistent with a declining and genetically depauperate population on Negros.

Interestingly, not only the historic museum specimens from Guimaras, but also the three from Masbate cluster close to the Panay haplotypes (Figure 2A). The Negros population is thus the only one clearly separated from the other insular populations (except for three individuals: PpNe11, 40, 57). Unfortunately, we could not genotype the museum specimens at the microsatellite loci; mtDNA, however, suggests genetic connectedness among Guimaras, Panay and Masbate. In the case of Guimaras, this is not surprising; only a narrow water channel of about 2 km separates nowadays this island from Panay (Figure 1). Obviously, this channel had not acted as such a strong geographic barrier as the sea between Panay/Guimaras and Negros. In the case of Masbate, it is noteworthy that the over water distance between Panay and Masbate is at least 35 km and thus longer than that between Negros and Panay. We hypothesize that the small islets lying in a row between Panay and Masbate could have acted as stepping stones facilitating dispersal.

Similarly to what was found for the Guimaras specimens, the Masbate specimens also carry unique haplotypes. Thus, Masbate also hosts an independent (yet closely related to Panay) lineage, which still survives with a few last individuals [19,22] or might have already got lost, if the population went extinct since the last census.

### *Penelopides manillae*

Both mtDNA and microsatellites indicate a separation between the two recognized subspecies *P. m. manillae* and *P. m. subniger*. However, divergence within one subspecies (*manillae*) is comparable to divergence between them (see Figure 2B and Table 5). In the haplotype network (Figure 2B), Pmm28 is an outlier. Although morphologically assigned to *P. m. manillae*, it clusters within the *P. m. subniger* haplogroup. The microsatellite profile of Pmm28 (coefficient of membership to *manillae* of about 90%; Figure 3B) indicates that this bird descends from a female ancestor that migrated from Polillo to Luzon generations ago.

Haplogroup I in the network (Figure 2B) is formed by Pmm17 and four museum specimens. Two of these museum specimens (ZMB 2000.22208, ZMB 32616) are labeled “Culion”, an island north of Palawan. For lack of any historical evidence for an occurrence of *P. manillae* on Palawan and its satellite islands, which, biogeographically, even do not belong to the Philippines, but to Borneo [4], we cannot rule out the possibility of these specimens having been transferred to Culion by humans. The correct geographic origin of these two individuals is thus considered uncertain. The other two (ZMB 9641, ZMB 21772) come from or around Manila. Two further museum specimens coming also from the Manila area (ZMB 21774, ZMB 2000.22207) cluster in haplogroup II. This implies that in the Manila area two divergent mitochondrial lineages coexist. Such a subdivision is not evident at the microsatellite level, though (Figure 3B). We cannot rule out the possibility that these results might be biased by our relatively small sample size that could have resulted in missing potentially extant intermediate haplotypes. On the other hand, given the strikingly contrasting pattern yielded by microsatellites, we tend to favor a scenario where two distinct mitochondrial lineages have coexisted for an extended period of time, either because of ancient larger population size or originating from two formerly separated populations within Luzon which later met and merged in the area around Manila.

In contrast, haplogroup III (Pmm24/25) stands out in both the mitochondrial and nuclear data sets, suggesting the occurrence of a further distinct genetic lineage on Luzon. *P. manillae* has a very wide distribution. Although encompassing islands such as Marinduque and Catanduanes which are as far away from the main island of Luzon as the Polillo Islands are, no further subspecies for those islands have been identified morphologically. Our data show that the lineage represented in our sampling by Pmm24/25 is genetically differentiated from both *P. m. manillae* (to which it was assigned in the first place) and *P. m. subniger*. More importantly, it is as distant from *P. m. manillae* as the latter subspecies is from *P. m. subniger*. While the instances of deviant mt haplotypes with inconspicuous microsatellite genotypes are indicative of historical gene flow (see above), the microsatellite genotypic compositions of Pms1, Pms9, Pms6, Pms3 might suggest ongoing gene flow, as these specimens presumably represent direct hybrids between a *P. m. subniger* female (inferred from the mtDNA type) and a *P. m. manillae* male. Although these samples originate from a non-breeding facility, we cannot exclude with certainty that these hybrids have been produced in captivity [25], such that our inference about naturally occurring gene flow and hybridization should be considered preliminary.

Apart from these hybrids, we hypothesize that *P. m. manillae* and *P. m. subniger* became separated when the sea level rose at the end of the Pleistocene and the Polillo Islands became disconnected from the main island of Luzon. While these islands were land-bridge connected to southern rather than central Luzon [2], they are nowadays about 20 km away from central Luzon. In spite of deforestation on Luzon, the coastline facing the Polillo Islands is still afforested, and migration between the islands would be still possible. Although tarictic hornbills are not renowned for long-distance dispersal, even to nearby offshore islands [52], migration events become the more likely, the more the pressure on the Polillo and Patnanungan populations increases due to logging, mining, and conversion of primary forest into coconut plantations [28]. Thus, gene flow might have been directed from the small Polillo Islands to the large island of Luzon by individuals attempting to escape the increasing human pressure. Such a scenario would explain the observed phylogeographic pattern; this, in turn, would be at odds with the theory of island biogeography that presumes large islands to serve as source for dispersal towards smaller islands [56,57].

The small population size of *P. m. subniger* as compared to that of *P. m. manillae* might be the result of the small size of the Polillo Islands combined with the loss of adequate habitat. This would explain the inferred recent bottleneck and the low values of genetic diversity of *P. m. subniger* (Table 5). Again, it should not be overlooked that our sample size for the subspecies is rather small.

### *Aceros waldeni*

In the haplotype network (Figure 2C), all three haplotypes of the five individuals from Guimaras cluster among the Panay haplotypes. Unfortunately, we do not have microsatellite data for these museum specimens; however, mtDNA (Table 5) suggests a certain degree of genetic connectedness between these two islands. The water channel between Guimaras and Panay is nowadays about 2 km wide and obviously did not act as an insurmountable barrier to gene flow. Between these two populations of *A. waldeni*, we did not find any major phylogeographic break. Nevertheless, each of the three haplotypes is unique. Thus, this island hosted independent (yet closely related) mitochondrial variants that probably got lost when the population went extinct.

However, despite having analyzed only a single museum specimen from Negros (BMNH 1897.5.13.471), similar to what was observed in *P. panini*, this individual indicates a separation between the Negros and the Panay/Guimaras population (Figure 2C). The same processes described for *P. panini* above may thus also have had an impact on *A. waldeni*. The Negros population is believed to be extinct or nearly so, as no recent sightings have been reported [EC pers. obs.]. Thus, this differentiated mitochondrial lineage has already gone extinct or is at least threatened with extinction, if any remaining hornbills are still alive.

## Conclusions

*P. manillae* is genetically more structured at the mtDNA level than *P. panini*. The latter is undergoing a very rapid continuing decline, associated with genetic depletion. Appreciable populations survived only on Panay and Negros. These two populations are genetically differentiated, as are those extinct or almost extinct from Guimaras and Masbate. This means, at least in the case of Guimaras, that part of the original genetic diversity of the species has already got lost.

At the microsatellite level, we found evidences of a recent bottleneck for *P. panini* from Negros, but also for *P. m. subniger* (Polillo Islands). While the latter population might be small in size because the islands it colonizes are also small, Negros is a large island and its population shrank solely because of loss of adequate habitat.

For *A. leucocephalus*, our data suggest that this species is (or was) genetically highly structured. *A. waldeni* has very rapidly decreased in number [12]. It survives with a substantial population size only on Panay, i.e., in the forest of the Central Panay Mountain Range [23]. *A. waldeni* generally exhibits lower genetic diversity values, both at microsatellites and mtDNA, than its less endangered sister species *A. leucocephalus*. Nevertheless, no sign for a recent bottleneck was found for *A. waldeni*. For the extinct population on Guimaras we could show that an independent (yet closely related) lineage probably has already got lost. From what we found in *P. panini*,we are inclined to hypothesize that the extinct or almost extinct population of *A. waldeni* on Negros is most likely genetically differentiated from that still extant on Panay. This hypothesis is further supported by the mt haplotype of the single museum specimen from Negros.

To sum up, the populations of the analyzed hornbill species are generally structured by the geological history of the area, although we found a few indications for across-island dispersal. By comparing patterns of genetic divergence and variability in both endangered Visayan hornbills species to that of their (less endangered) sister taxa, we revealed lower genetic diversity associated with their dramatic population decline and the extinction of genetically differentiated populations. Conservation efforts maintaining the surviving wild birds of virtually extinct populations are therefore particularly important, as well as the preservation of their genetic potential in captivity. This applies especially to the potentially still existing populations of *A. waldeni* on Negros and *P. panini* on Masbate.

## Competing interests

The authors declare that they have no competing interests.

## Authors’ contributions

SS designed the study, conducted most of the lab work, carried out the sequence alignments, performed most of the genetic analyses, and drafted the manuscript. VK participated in the discussion of results, performed part of the population genetics analyses and the phylogenetic analyses for the online supplement, wrote the corresponding text parts, and critically revised the manuscript. KH and UK performed part of the lab work. EC organized most of the sampling, participated in designing the study, and revised the manuscript. RT supervised and participated in designing the study, participated in the discussion of results, and critically revised the manuscript. All authors read and approved the final manuscript.

## Supplementary Material

Additional file 1 Note on the museum label of ZMB 21773. Click here for file
