# Supplementary material for: Mitochondrial control region I and microsatellite analyses of endangered Philippine hornbill species (Aves; Bucerotidae) detect gene flow between island populations and genetic diversity loss
Source: BMC Evol Biol. 2012 Oct 12;12:203. doi: 10.1186/1471-2148-12-203 (PMC3532089; doi:10.1186/1471-2148-12-203)
Supplement: Additional file 1 — Note on the museum label of ZMB 21773. [file 1471-2148-12-203-S1.doc]

**Note on the museum label of ZMB 21773**

The following analyses were carried out to determine the closest relative(s) of the museum specimen ZMB 21773. This bird was collected by Adolf Bernhard Meyer in February 1872 on Luzon (as reported in the museum voucher). Based on geographic considerations alone, this adult male (red framed and photographed in Figure 1) was hence first identified and labeled as *P. manillae*, even though its plumage pattern is more *affinis* than *manillae*-like. It possesses morphological features that fit best *P. affinis affinis*, a taxon occurring in Mindanao: The tail band (9.4 cm at the central feather) is wider than that described for *P. m. manillae* [1] and even wider than that of all the *P. m. subniger* individuals used in this study (4.3-8.2 cm). The bill is horn-colored with a black patch at the base of the upper non-ridged mandible (Figure 1) as described for *P. affinis* [1]. Based on morphological features, the label on the voucher of ZMB 21773 was later corrected to *P. affinis*.

Our phylogenetic analyses were run on a data set including all *P. manillae*, two additional *P. affinis* samples (Pa) from Mindanao (collected in the Malagos Garden Resort, Davao City), and with one sample of *A. leucocephalus* and *A. waldeni* each as outgroup. We used JModelTest [2] to determine the model of sequence evolution that best fit our data. We then used the JModelTest output for the Maximum Likelihood (ML) and Bayesian searches. ML analyses were carried out with the fastDNAml 1.2.2 program [3] available on the Mobyle portal (<http://mobyle.pasteur.fr/cgi-bin/portal.py>) with 1,000 bootstrap replicates. The Bioportal of the University of Oslo (Norway) [4] was used to run the Bayesian searches. These were run in MrBayes 3.0 [5] for 4,000,000 generations with a sampling frequency of 100 generations. We ran one cold and three heated Markov chains and two independent runs. To ascertain if the Markov chains had reached stationarity, we created a cumulative split frequency plot of the likelihood scores of sampled trees against generation time using AWTY online software [6]. Trees generated before the stationarity phase were discarded as burn-in (first 10% of the sampled trees), and posterior probability values for each node were calculated based on the remaining 90% of sampled trees. These trees were used to construct a 50% majority rule consensus tree using PAUP* 4.0b10 [7]. PAUP was also used to analyze data by Maximum Parsimony (MP) with 1,000 bootstrap replicates (heuristic searches, ACCTRAN character-state optimization, 100 random stepwise additions, TBR branch-swapping algorithm) [8,9]. Data are deposited in TreeBase under the submission ID 13354.

We found ZMB 21773 [GenBank: JX273975] clustering robustly with the two individuals of *P. affinis* (see Figure) [GenBank: JX273976-JX273977]. This individual was thus excluded from the analyses presented in Table 3 and in Figure 2B. In the phylogenetic analyses, ML, Bayesian (both based on the General Time-Reversible with Gamma-distributed rate heterogeneity and Invariant sites as selected by JModelTest; GTR+ Γ + I) and MP searches yielded virtually indistinguishable topologies; Figure 1 shows the ML topology and summarizes the results from the other two phylogenetic methods employed in the study. The main supported clades correspond to the major haplogroups identified in the network of Figure 2B. These analyses also strongly place *P. affinis* within *P. manillae*, rendering the latter taxon paraphyletic.

Given the morphology and mtDNA haplotype, we confirm the previous correction of the taxonomic status of the specimen (i.e., its assignment to *P. affinis*. In the light of our analysis, however the geographic origin of ZMB 21773 given in the museum voucher (Luzon) appears potentially erraneous. In our opinion, one cannot rule out the possibility that this bird was collected on a market on Luzon after having been captured and brought there from somewhere in Mindanao, where *P. affinis* *affinis* naturally occurs.

1. Kemp AC: *The Hornbills.* Oxford: Oxford University Press; 1995.
2. Posada D: **jModelTest: phylogenetic model averaging.** *Mol Biol Evol* 2008, **25(7)**:1253-1256.
3. Olsen GJ, Matsuda H, Hagstrom R, Overbeek R: **fastDNAml: a tool for construction of phylogenetic trees of DNA sequences using maximum likelihood.** *Comput Appl Biosci* 1994, **10(1)**:41-48.
4. Kumar S, Skjæveland Å, Orr RJS, Enger P, Ruden T, Mevik BH, Burki F, Botnen A, Shalchian-Tabrizi K: **AIR: a batch-oriented web program package for construction of supermatrices ready for phylogenomic analyses.** *BMC Bioinformatics* 2009, **10**:357.
5. Ronquist F, Huelsenbeck JP: **MrBayes 3: Bayesian phylogenetic inference under mixed models.** *Bioinformatics* 2003, **19(12)**:1572-1574.
6. Nylander JAA, Wilgenbusch JC, Warren DL, Swofford DL: **AWTY (are we there yet?): a system for graphical exploration of MCMC convergence in Bayesian phylogenetics.** *Bioinformatics* 2008, **24(4)**:581-583.
7. Swofford DL: *Paup*4.0b10. Phylogenetic analysis using parsimony (and other methods).* Sunderland: Sinauer; 2003.
8. Farris JS: **Methods for computing Wagner trees.** *Syst Zool* 1970, **19(1)**:83-92.
9. Hendy MD, Penny D: **Branch and bound algorithms to determine minimal evolutionary trees.** *Math Biosci* 1982, **59(2)**:277-290.


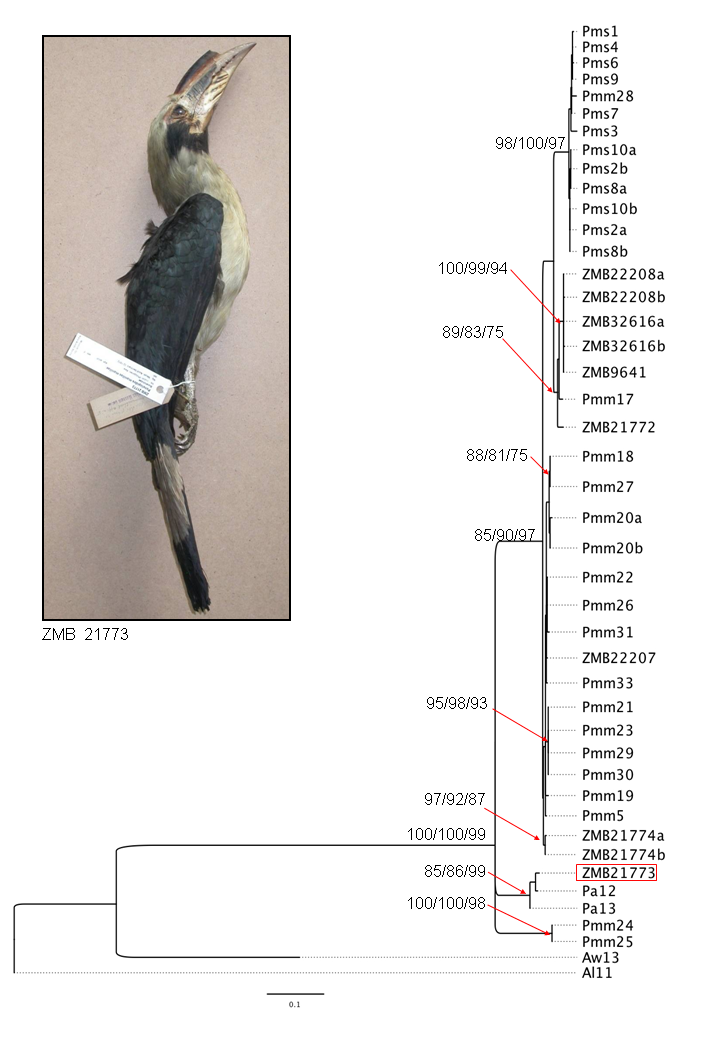
 Maximum Likelihood tree for the *Penelopides manillae manillae* (Pmm), *P. m. subniger* (Pms) and *P. affinis* (Pa) haplotypes found in the study.

The tree is based on the GTR + Γ +I model (α = 0.136; I = 0.218) with *Aceros waldeni* (Aw) and *A. leucocephalus* (Al) as outgroups. Numbers at nodes are bootstrap support values for ML and MP searches (1000 replicates; first and third value, respectively) and posterior probabilities for the Bayesian search (second value). Only support values ≥ 75% are reported. For heteroplasmic individuals, both haplotypes are shown, separated by a and b after the individual’s unique number. ZMB 21773 (red framed, photographed) is a museum specimen of alleged Luzon origin and labeled as *P. manillae* (as reported in the museum voucher), but which possesses plumage and bill patterns best fitting those of *P. affinis*, a species confined to Mindanao.
